# Genome-Wide Identification and Functional Divergence of the Chloride Channel (CLC) Gene Family in Autotetraploid Alfalfa (*Medicago sativa* L.)

**DOI:** 10.3390/ijms262311442

**Published:** 2025-11-26

**Authors:** Yanjun Fang, Guangzhi Jiang, Pingping Du, Jiayin Wang, Huan He, Hongfei Li, Hongbin Li, Fei Wang, Quanliang Xie

**Affiliations:** Xinjiang Production and Construction Corps Key Laboratory of Oasis Town and Mountain-basin System Ecology, Key Laboratory of Xinjiang Phytomedicine Resource Utilization, Ministry of Education, College of life Sciences, Shihezi University, Shihezi 832003, China; m17503889050@163.com (Y.F.); jiangguangzhi2024@163.com (G.J.); dopingping@126.com (P.D.); wjyinee@163.com (J.W.); he_huan026@163.com (H.H.); 15001612743@163.com (H.L.); lihb@shzu.edu.cn (H.L.)

**Keywords:** alfalfa, chloride channel protein, abiotic stress, chloride ions

## Abstract

Chloride channel proteins (CLCs) are essential anion transporters involved in plant growth, osmotic regulation, and ion homeostasis. However, their genome-wide characterization in tetraploid alfalfa (*Medicago sativa* L.) remains unexplored. In this study, a total of 35 CLC family members were identified and underwent comprehensive bioinformatic analyses. Phylogenetic and structural analyses divided them into six subfamilies and two subclasses based on conserved residues such as GxGIPE. Members within the same subclass shared conserved domains and similar motif patterns. Analysis of duplication events indicated that 48 segmental duplications were the primary driving force behind the expansion of this gene family. Promoter analysis revealed abundant light, hormone, and stress-responsive cis-elements, suggesting multiple regulatory functions. Gene expression profiling demonstrated that salt, drought stress, and ABA treatment significantly induced the expression levels of some genes. Among them, *MsCLC2* and *MsCLC18* from Group c exhibited more than fivefold upregulation under both salt and drought stress, significantly higher than other members. Subcellular localization confirmed *MsCLC18* on the plasma membrane, potentially regulating Cl^−^ efflux through a Cl^−^/H^+^ antiporter mechanism to alleviate Cl^−^ toxicity. These findings provide a theoretical foundation for the function study of *CLC* genes in alfalfa and offer new insights into the molecular evolution of polyploid plants under abiotic stress.

## 1. Introduction

Soil salinization has become an increasingly severe global challenge, threatening agricultural security [1]. Currently, it impairs over 20% of the world’s arable land and 33% of irrigated farmland, with the extent increasing by 1.5 million hectares each year [2,3]. Soil salinization is primarily caused by various soluble salts such as chlorides, sulfates, and nitrates [4]. Among these, sodium chloride is the predominant salinizing agent in agricultural soils. It is also the key factor responsible for salt ion toxicity in plants [5].

Currently, extensive research has focused on Na^+^ toxicity and its adaptive mechanisms in plants; Cl^−^ toxicity has received far less attention [6]. As an essential macronutrient [7,8], Cl^−^ plays vital roles in plant growth, leaf development, photosynthesis, osmotic homeostasis, and water balance [9,10,11]. However, its excessive accumulation induces a range of detrimental effects, including disrupted nutrient uptake, leaf damage, inhibited photosynthesis, and oxidative stress [12,13]. Notably, legumes exhibit heightened sensitivity to chloride ions, such as mung beans, *Glycine max*, and *Lotus corniculatus* [14,15,16,17,18,19], as well as woody plants including *Malus hupehensis*, *Vitis vinifera*, and *Diospyros virginiana* [20,21,22]. Therefore, understanding how plant cells regulate chloride ion homeostasis is crucial for deciphering salt tolerance mechanisms and breeding resilient crops.

CLC (chloride channel) is a crucial anion transporter. Its first family gene, CLC-0, was cloned from the electric organ tissue of *Torpedo marmorata* in 1990 [23]. Subsequently, homologous genes were successively identified in various plants, including *Arabidopsis thaliana*, *Triticum aestivum*, *Solanum lycopersicum*, and *Nicotiana tabacum* [24,25,26,27]. Studies indicate that these proteins are widely distributed across both biological membranes and organellar membranes [28]. Eukaryotic CLC proteins contain a conserved voltage-gated chloride channel (Voltage-gate CLC) domain and two conserved Cystathionine beta synthase (CBS) domains [29,30]. Furthermore, CLC proteins possess three conserved residues crucial for anion selectivity: GxGIPE (I), GKxGPxxH (II), and PxxGxLF (III). The x residue in GxGIPE (I) determines anion specificity: when x is proline (P), the protein preferentially transports NO_3_^−^, whereas when x is serine (S), it preferentially transports Cl^−^. When the x in the conserved region PxxGxLF (II) is a conservative-gated glutamate (E) and the fourth residue in the conserved region PxxGxLF (III) is proton glutamate (E) residue, the protein exhibits antiporter activity; if other amino acids are present at these positions, it may function as an ion channel [31]. These functional predictions based on conserved residues provide important insights into the potential role of CLC proteins in ion homeostasis.

Studies have demonstrated that the *CLC* gene family is a crucial regulator of Cl^−^ homeostasis under salt stress [32]. Its mechanism of action primarily involves two pathways: Cl^−^ efflux and vacuolar compartmentalization. In cotton, *GhCLCc-1* enhances salt tolerance by mediating Cl^−^ efflux and reducing its accumulation in plants [33]. In *Arabidopsis thaliana*, *AtCLCf* translocates from the Golgi apparatus to the plasma membrane under high salt stress to participate in Cl^−^ extrusion, thereby increasing salt tolerance [34]. In *Malus hupehensis*, *MhCLC-c1* reduces cell death by decreasing intracellular Cl^−^ content [20]. On the other hand, some *CLC* genes alleviate Cl^−^ toxicity through ion compartmentalization. For instance, *Glycine max GmCLC1* enhances salt tolerance by sequestering Cl^−^ in root vacuoles, reducing Cl^−^ accumulation in aerial parts [35]; *Oryza sativa OsCLC-1/2* improves chloride tolerance by compartmentalizing Cl^−^ in vacuoles [36]. Beyond their salt tolerance functions, *CLC* genes also play important roles in maintaining cell turgor, regulating stomatal movement, ion homeostasis, enhancing drought and cold tolerance, and heavy metal detoxification [27,37,38,39,40].

Alfalfa is a globally cultivated leguminous plant crucial for livestock production, agricultural sustainability, and ecological conservation [41,42]. Among these, the tetraploid ‘Xinjiangdaye’ alfalfa, as one of the primary forage crops in Northwest China, exhibits high stress tolerance and adaptability [43]. However, the region suffers from scarce water and soil resources, with salinization and drought stress being the primary factors limiting the growth and promotion of ‘Xinjiangdaye’ alfalfa [44]. Given the documented sensitivity of legumes like *Glycine max* and *Lotus japonicus* to Cl^−^ toxicity [15,16], alfalfa is likely similarly vulnerable. This study focuses on autotetraploid alfalfa due to its distinct advantages in genetic architecture and stress resistance mechanisms. Compared to diploid plants, genome duplication in tetraploid alfalfa creates gene redundancy. This enhances traits including growth, photosynthetic efficiency, and environmental adaptability, while also promoting expansion and functional differentiation of stress-related gene families [45,46]. These changes establish a broader genetic variation base and enhance stress response potential [47]. The ‘Xinjiangdaye’ cultivar, a sequenced autotetraploid, demonstrates high biomass production and strong ecological adaptability [48]. However, the *CLC* gene family, important for stress response, remains unstudied in tetraploid alfalfa.

This study presents the first genome-wide identification and characterization of the *CLC* gene family in the autotetraploid alfalfa ‘Xinjiangdaye’. Comprehensively investigated the chromosomal distribution characteristics, phylogenetic relationships, conserved motifs and domain organization, gene structure features, gene duplication events, collinearity relationships, and cis-acting element analysis of *MsCLCs* family members. Based on promoter cis-element analysis and transcriptome data under salt stress, key genes potentially associated with stress resistance were screened from each subfamily, and the expression patterns of these seven candidate genes under salt, drought, and ABA treatments were further validated using qRT-PCR technology. Additionally, subcellular localization of *MsCLC18* was investigated using an onion epidermal cell transient transformation system. This research systematically analyzed the bioinformatic characteristics of the *MsCLCs* gene family, highlighting the distinctive advantages of the tetraploid alfalfa genetic resources in stress resistance studies. It also provides a solid foundation for subsequently cloning key genes and further exploring their biological functions under osmotic stress conditions such as high salinity and drought, ultimately offering an important theoretical support for breeding new forage varieties with enhanced stress tolerance.

## 2. Results

### 2.1. Identification and Classification of the MsCLCs Gene Family

**Figure 1:**
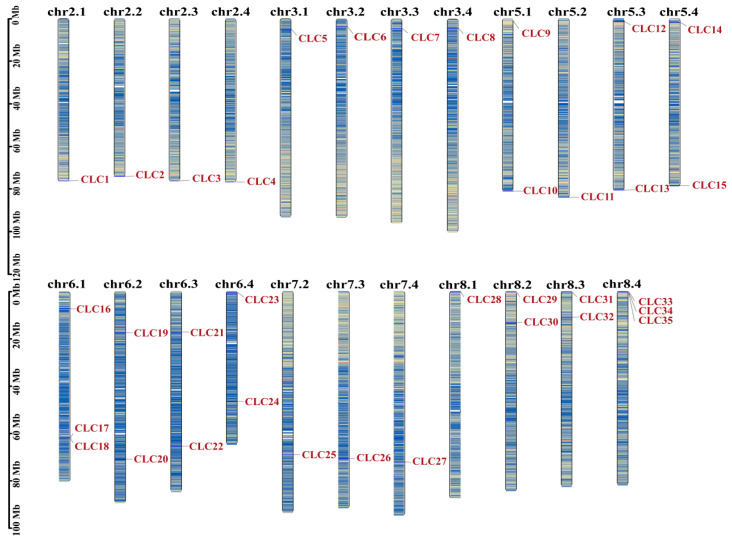
Schematic diagram of the chromosomal distribution of *MsCLC* genes in *Medicago sativa*. The vertical bars represent the chromosomes of *Medicago sativa*, and a scale for chromosome length is shown on the left. Chromosome numbers are shown in black at the top, while *MsCLC* genes are marked in dark red. The color gradient from red to blue represents the gene density from high to low.

A total of 35 *CLC* genes were identified in alfalfa through Blastp and Hmmer alignments using *Arabidopsis* CLC protein sequences as queries against the ‘Xinjiangdaye’ genome database. The 35 *MsCLC* genes were unevenly distributed across 23 chromosomes. They were subsequently renamed *MsCLC1* to *MsCLC35* according to their positional arrangement on the chromosomes (Figure 1). Essential information regarding the chromosomal locations and chromosome lengths of the *MsCLCs* gene family in alfalfa is provided in Table S1.

Physicochemical characterization of the *MsCLCs* gene family is presented in Tables S2 and S3. The MsCLC proteins ranged from 625 to 1436 amino acids in length, with molecular weights between 68.4 and 154.9 kDa and theoretical isoelectric points (pI) ranging from 5.54 to 9.13, including 16 acidic and 19 basic proteins. The instability index (30.57–42.07) and aliphatic index (95.75–112.05) suggested that most MsCLCs were stable and flexible. GRAVY values (−0.044–0.563) indicated that the majority were hydrophobic, with only three being hydrophilic. Subcellular localization predictions indicate that 34 MsCLC proteins were localized to the plasma membrane. Among these, MsCLC5-8 were also localized to the mitochondria, while only MsCLC20 was predicted to reside in the chloroplast. Online prediction using TMHMM Server 2.0 revealed that the MsCLC protein family contained 7 to 19 transmembrane domains (Figure S1 and Table S3). This structural feature aligned with the attributes of membrane proteins and implied their potential role as ion transporters. Secondary structure predictions showed that random coils and α-helices constituted the main secondary structures of the MsCLC proteins, with extended strands and β-sheets sporadically distributed throughout the proteins (Figure S2 and Table S3).

### 2.2. Phylogenetic Analysis and Multiple Sequence Alignment Analysis of MsCLC Proteins

**Figure 2:**
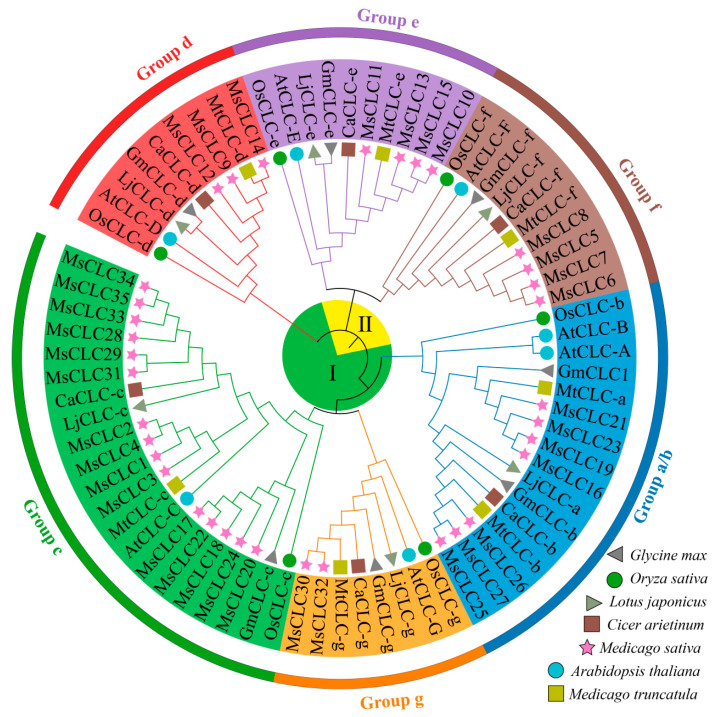
Phylogenetic tree of *CLC proteins* in *Medicago sativa* versus other species. The improved trees were constructed by adjacency method, including *Medicago sativa* (MsCLCs), *Arabidopsis thaliana* (AtCLCs), *Medicago truncatula* (MtCLCs), *Oryza sativa* (OsCLCs), *Glycine max* (GmCLCs) and other species.

To explore the evolutionary relationships of MsCLC proteins. A phylogenetic analysis was performed using 74 CLC sequences from seven species, including *Medicago sativa*, *Oryza sativa*, *Arabidopsis thaliana*, *Medicago truncatula*, *Glycine max*, *Lotus japonicus*, and *Cicer arietinum* (Table S4). The MsCLCs proteins were classified into six subfamilies, designated Group a/b through Group g (Figure 2). Specifically, MsCLC16, 19, 21, 23, 25, 26, and 27 belonged to Group a/b; MsCLC30 and MsCLC32 to Group g; MsCLC1–4, 17, 18, 20, 22, 24, 28, 29, 33, 34, and 35 to Group c; MsCLC9, 12, and 14 to Group d; MsCLC10–13 to Group e; and MsCLC5–8 to Group f. Among these, Group c was the largest subfamily with 14 members, while Group g was the smallest, containing only two members. Overall, proteins within the same subfamily exhibited close phylogenetic relationships, suggesting they may share similar biological functions.

Multiple sequence alignment of the 35 MsCLCs protein sequences from alfalfa was performed, and three conserved residues were analyzed using MAST (Figure S3). Based on these three conserved residues, the CLC proteins could be further divided into two subclasses. Group a/b, Group g, Group c, and Group d all contained the conserved motifs GxGIPE (I), GKxGPxxH (II), and PxxGxLF (III), and were classified as Subclass I. In contrast, Group e and Group f lacked these three conserved residues and were categorized as Subclass II.

### 2.3. Conserved Motifs, Domains, and Gene Structures of MsCLC Genes Family

**Figure 3:**
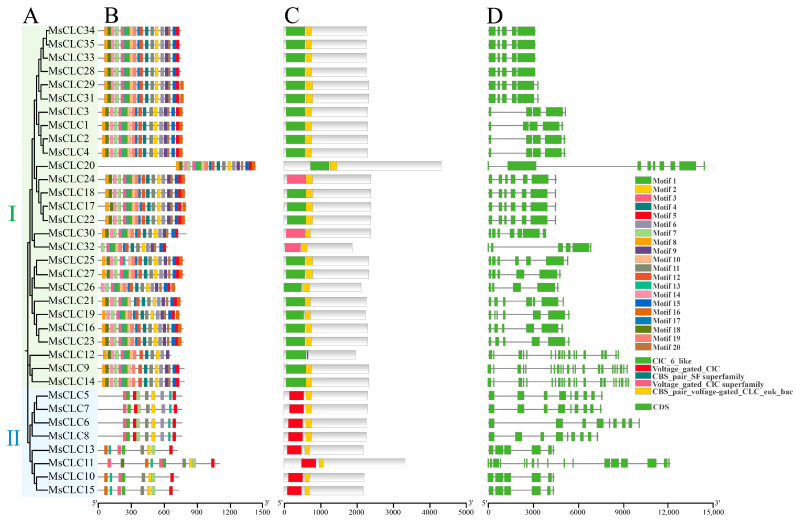
The evolutionary tree, motifs, domains, and gene distribution of MsCLC proteins. (**A**) The evolutionary tree of MsCLCs (green section is subclass I, blue section is subclass II). (**B**) Distribution of conserved motifs in MsCLCs. (**C**) Distribution of conserved domains in MsCLCs. (**D**) Gene structure of MsCLCs, including introns (black lines) and CDS (green rectangles).

To further investigate the evolutionary relationships within the *MsCLCs* gene family, a comprehensive visualization analysis was performed on the conserved motifs, domains, and gene structures of its 35 members (Figure 3). Motif analysis identified a total of 20 conserved motifs (Figure 3B and Figure S4). All MsCLCs proteins were found to contain Motif 2, Motif 3, Motif 7, Motif 11, and Motif 13, indicating high conservation of these motifs within the gene family and suggesting their critical functional importance. Furthermore, significant differences in motif composition were observed between the two subclasses. MsCLC proteins within the same subclass exhibited similar numbers and types of motifs. For instance, all members of Subclass II possessed Motif 1, Motif 2, Motif 3, Motif 5, Motif 7, Motif 11, and Motif 13, indicating their distinct evolutionary history and potential functional specialization.

Domain analysis revealed that all MsCLCs proteins contain the CLC domain and CBS_pair domain characteristic of the CLC family (Figure 3C). However, the CLC6-like, voltage-gated CLC superfamily, and CBS-pair SF superfamily domains were present only in Subclass I, while the voltage-gated CLC domain was found exclusively in Subclass II. Differences in domain composition reflect potential distinct functional divergences among subclasses during evolution.

Gene structure analysis showed substantial variation in exon numbers among the *MsCLC* genes in alfalfa, ranging from 5 to 22. Within Group d, genes *MsCLC9*, *MsCLC12*, and *MsCLC14* contained the highest number of exons. In contrast, genes *MsCLC1–4* in Group c possessed the fewest, with only 5 exons each (Figure 3D).

These results demonstrated that each subclass possessed a unique domain composition and shared similar conserved motifs, indicating that while the functional roles of *CLC* gene family members in alfalfa had remained relatively conserved during evolution, a degree of functional divergence had also occurred.

### 2.4. Gene Duplication Events and Colinearity Analysis of the MsCLCs Gene Family

**Figure 4:**
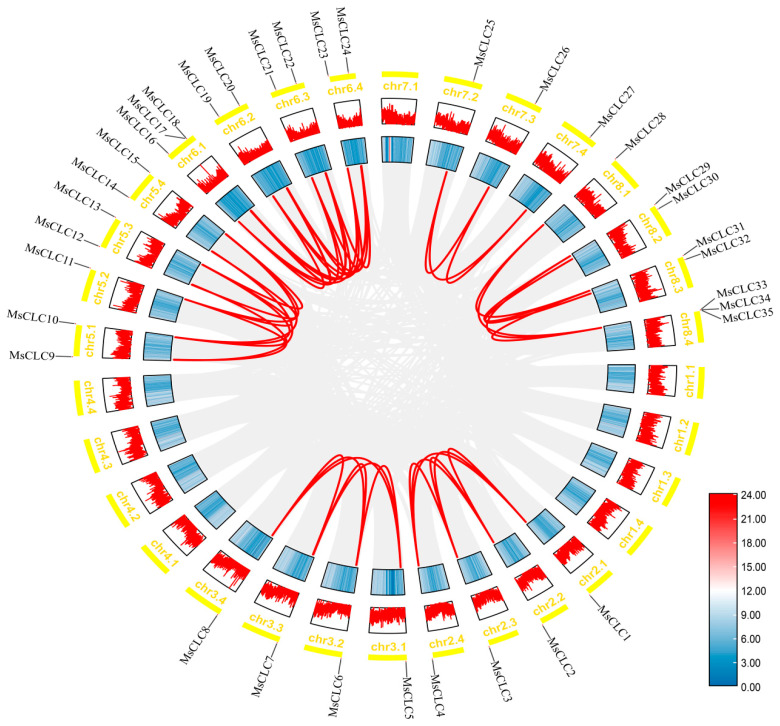
A circular figure showing *MsCLC* genes’ collinearity. The chromosomal locations of the *MsCLC* genes are shown on the outer side, with yellow boxes indicating different chromosomes. The outer ring of blue and red represents gene density. The fragments of the duplicated *MsCLC* genes are connected by red curves, and genomic wide collinear blocks are used as the background (gray).

**Figure 5:**
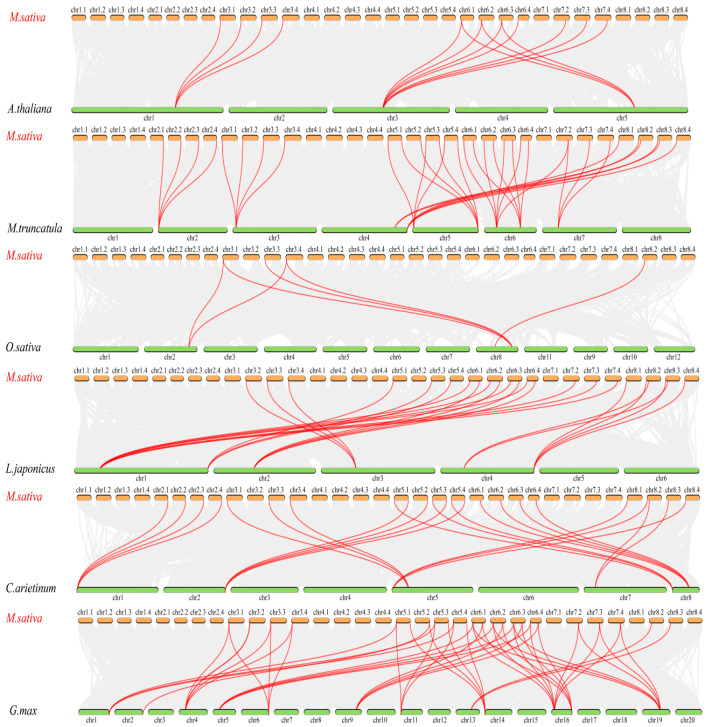
The homologous relationship of CLC members between *Medicago sativa* and six plant species; the gray lines (background) indicate the syntenic regions of *Medicago sativa* with other plant genomes; the red lines highlight the homologous *MsCLC* gene pairs.

To further investigate potential gene duplication events among *MsCLC* genes, we performed an intra-species collinearity analysis based on the genome database of the ‘Xinjiangdaye’ variety (Figure 4). The results indicated that no tandem duplication events were detected among the 35 MsCLCs members. However, a total of 48 homologous gene pairs generated by segmental duplication events were identified, such as *MsCLC1* and *MsCLC4* located on different chromosomes, suggesting that segmental duplication played a major role in the expansion and evolution of the *MsCLC* genes. We calculated the Ka/Ks ratios for homologous *MsCLC* gene pairs to assess selective pressure (Table S5). All ratios were less than 1, indicating that the *MsCLC* genes family had undergone purifying selection. This strong evolutionary constraint underscores a high degree of functional conservation, suggesting these genes play a critical biological role in plants. Furthermore, to explore the phylogenetic relationships of the *CLC* gene family across different plant species and to clarify the contribution of polyploidization to the expansion of this gene family, we conducted an inter-species collinearity analysis between *Medicago sativa*, *Oryza sativa*, *Arabidopsis thaliana*, *Medicago truncatula*, *Glycine max*, *Lotus japonicus*, and *Cicer arietinum*. (Figure 5 and Table S6). The results revealed 40, 33, 23, 23, 14, and 6 homologous *CLC* gene pairs between *Medicago sativa* and *Glycine max*, *Medicago truncatula*, *Lotus japonicus*, *Cicer arietinum*, *Arabidopsis thaliana*, and *Oryza sativa*, respectively. The number of homologous gene pairs between alfalfa and legume species was significantly higher than that with non-legume species, indicating a closer phylogenetic relationship between alfalfa and other legumes and a higher evolutionary conservation of *CLC* genes within legume plants. Moreover, a single homologous gene in *Medicago truncatula* (a diploid species) could correspond to up to four *CLC* genes in alfalfa (a tetraploid species) (Table S6). This finding directly demonstrated the key role of polyploidization in the expansion of the *CLC* gene family in alfalfa.

### 2.5. Promoter Cis-Regulatory Elements Analysis of MsCLCs Gene Family

**Figure 6:**
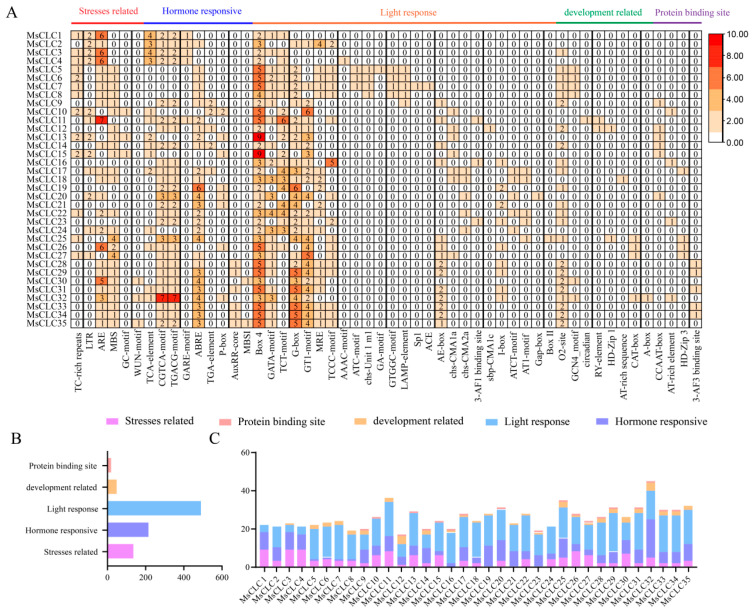
Classification and statistics of cis-acting elements in the *MsCLCs* gene family. (**A**) Classification heat map of cis-acting elements, which is divided into 5 categories, and the numbers in the heatmap represent the number of cis-acting elements. (**B**) Classification of all cis-acting elements in the *MsCLCs* gene family. Different colors represent different species. (**C**) Statistics of cis-acting elements for each *MsCLC* genes. Different colors in the stacked chart represent different species.

To predict the functions and regulatory mechanisms of *MsCLC* genes, potential cis-acting elements within the 2 kb upstream promoter regions of these genes were identified and analyzed using PlantCARE. The results are shown in Figure 6 and Figure S5. A total of 52 types of cis-acting elements were identified in the promoter regions of *MsCLC* genes. These include 25 types related to light responsiveness, 6 types associated with stress responses covering defense and stress, drought, low-temperature, anaerobic induction, and hypoxia-inducible elements, and 9 types involved in hormone responses such as salicylic acid, methyl jasmonate, gibberellin, abscisic acid, auxin, and flavonoid biosynthesis regulatory elements. Furthermore, 8 types were linked to tissue development and regulation, including zein metabolism regulation, endosperm expression, palisade mesophyll cell differentiation, meristem expression, circadian control, and seed-specific regulation, and 4 types were protein-binding elements, including protein-binding sites, ATBP-1 binding sites, and elicitor-mediated activation elements (Figure 6A). Within the *MsCLCs* family, light-responsive elements were the most abundant with 491 instances, followed by hormone-responsive elements and stress-responsive elements with 213 and 134 instances, respectively (Figure 6B). Among the family members, *MsCLC32* contained the highest number of cis-acting elements, while *MsCLC12* contained the fewest (Figure 6C). These results suggested that the *MsCLCs* gene family may play important roles in various physiological and metabolic processes, including light response, hormone signaling, and abiotic stress responses.

### 2.6. Analysis of the Expression Patterns of MsCLC Genes Under Different Abiotic Stress Treatments

To investigate the responses of different *MsCLC* subfamilies to salt, drought, and exogenous ABA, this study selected representative genes from each subfamily. Based on transcriptome data (PRJNA450305) and incorporating the adversity response element (ABRE) identified through promoter cis-element analysis, representative genes were ultimately selected from each subfamily for subsequent experiments (Figure S6). Given that Group c contained the largest number of *MsCLC* genes and its members might function as Cl^−^ antiporters, two genes were selected from this group. Ultimately, *MsCLC2, 8, 11, 14, 18, 27*, and *30* were chosen for RT-qPCR validation under salt, drought, and ABA treatments.

**Figure 7:**
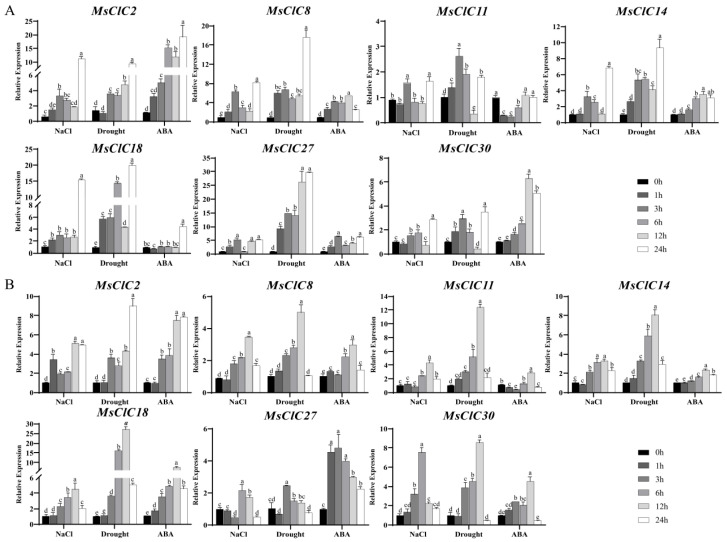
Expression Pattern Analysis of Partial *MsCLC* Genes in *Medicago sativa* Under Osmotic Stress and Exogenous ABA Treatment. (**A**) Gene expression patterns in leaves under treatment conditions. (**B**) Gene expression patterns in roots under treatment conditions. The data in the figure are the mean ± SDs of triplicate replicates, and the different letters indicate significant differences (*p* < 0.05).

The results revealed that under salt stress (Figure 7), the expression patterns in leaves generally followed an “increase-decrease-reincrease” trend, peaking at 24 h, except for *MsCLC18* which showed an initial rise at 1 h followed by stable expression before reaching its peak at 24 h (Figure 7A). In roots, *MsCLC8*, *11*, *14*, *18*, and *30* exhibited a consistent pattern of gradual increase followed by decrease; *MsCLC30* peaked at 6 h, while the others reached their peak expression at 12 h. *MsCLC2* displayed an “increase-decrease-reincrease” trend, peaking after 12 h. In contrast, *MsCLC27* expression decreased at 3 h, then significantly increased to its peak at 6 h before declining again (Figure 7B).

Under drought stress, *MsCLC2* and *MsCLC27* in leaves exhibited a consistent expression pattern, steadily increasing and peaking at 24 h. The remaining genes followed an “increase-decrease-reincrease” trend; while *MsCLC11* reached its peak at 3 h, all others peaked at 24 h (Figure 7A). In roots, *MsCLC8*, *11*, *14*, *18*, and *30* showed a continuous increase, peaking at 12 h before declining. *MsCLC2* expression increased at 3 h, decreased at 6 h, and then gradually rose again, reaching a significant peak at 24 h. *MsCLC27* expression rose rapidly to its peak by 3 h and subsequently decreased (Figure 7B).

Under ABA treatment, *MsCLC8, 14*, and *30* in leaves displayed a similar expression pattern, continuously increasing to a peak at 12 h before declining. *MsCLC2* expression gradually increased and peaked at 24 h. *MsCLC27* followed a “decrease-increase-redecrease” trend, with peaks observed at 3 h and 24 h. *MsCLC18* showed little fluctuation within the first 12 h and reached its peak at 24 h. *MsCLC11* expression was initially downregulated but returned to control levels by 12 h (Figure 7A). In roots, *MsCLC1*, *8*, *14*, *18*, and *30* shared a consistent expression pattern, increasing steadily and peaking at 12 h. *MsCLC27* expression peaked at 3 h before decreasing. *MsCLC11* expression initially decreased, reached its lowest point at 3 h, and then increased to a peak at 12 h (Figure 7B).

The results demonstrated that *MsCLC* genes were induced by salt, drought, and ABA treatments, with the most significant expression changes observed under salt and drought stresses. Among them, *MsCLC2* and *MsCLC18* from Group c exhibited particularly notable responses, showing more than five-fold upregulation in expression. It was noteworthy that *MsCLC18* displayed a “root-to-leaf temporal response” pattern under salt stress: it responded rapidly in the roots, reaching peak expression at 12 h, while its expression in leaves was delayed, peaking at 24 h. In conclusion, *MsCLCs* genes, especially *MsCLC18*, were likely to play important regulatory roles in alfalfa’s response to osmotic stress.

### 2.7. Subcellular Localization Analysis

**Figure 8:**
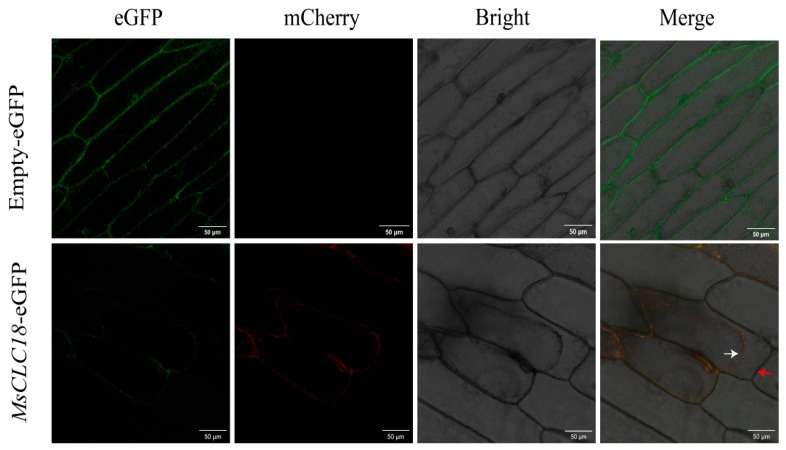
Subcellular localization of MsCLC18 protein. The image from left to right represents green fluorescent protein (GFP), cell membrane marker, bright field, and mixed field from the same sample. The white arrows refer to the cell membrane and the red arrows refer to the cell wall. bar = 50 μm.

Subcellular localization predictions indicated that 34 members of this family were localized to the plasma membrane. The *MsCLC18* gene was selected as a representative for experimental subcellular localization analysis due to its significant and specific expression pattern under stress conditions. In the control group, onion epidermal cells transiently transformed with the empty vector (pCAMBIA1300-eGFP) exhibited green fluorescent signals in the nucleus, plasma membrane, and cytoplasm. In contrast, the green fluorescent signal from the transiently expressed MsCLC18-eGFP fusion protein was detected exclusively in the plasma membrane and overlapped with the red membrane-specific marker (Figure 8). This result experimentally confirms the plasma membrane localization of the MsCLC18 protein. These findings provided key experimental support for the predicted plasma membrane localization of the majority of *MsCLCs* family members.

## 3. Discussion

Alfalfa, as an important forage crop widely cultivated worldwide, holds significant economic and ecological value [49]. However, increasing soil salinization has made salt stress a leading abiotic constraint on alfalfa growth and development [50,51,52]. Under high-salinity, CLC proteins regulate the accumulation of anions in plants, thereby conferring salt tolerance [53]. Notably, legume research has predominantly focused on nitrogen fixation and symbiosis, with limited attention given to ion transport mechanisms [17]. This study aims to identify and characterize the *CLC* gene family in alfalfa, providing a theoretical basis and candidate gene resources for breeding salt-tolerant alfalfa varieties.

In this study, a total of 35 *MsCLC* genes were identified and found to be unevenly distributed across 23 chromosomes (Figure 1). Phylogenetic analysis integrating *CLC proteins* from *Arabidopsis thaliana*, *Oryza sativa*, *Medicago truncatula*, *Glycine max*, *Cicer arietinum*, and *Lotus japonicus* classified the MsCLCs into six subfamilies (Figure 2). Further conserved residue analysis categorized them into two subclasses based on the presence or absence of three conserved residues critical for CLC protein function. This classification is consistent with CLC categorization in other species. For instance, the seven AtCLC family members in *Arabidopsis thaliana* are divided into seven subfamilies and two subclasses [31], while wheat TaCLC family members are also classified into two subclasses [26]. Moreover, variations in conserved residues suggest potential functional divergence among these proteins [31,32]. Analysis of key residues across different subfamilies revealed significant functional differentiation in ion transport among MsCLCs family members, while maintaining high evolutionary conservation (Table S7). Specifically, Group e and Group f lack typical functional residues and may function as non-selective ion channels. Groups c, d, and g, with serine (S) at the x position in conserved residue (I), preferentially mediate Cl^−^ transport. Groups a/b, with proline (P) at the x position in conserved residue (I), preferentially transport NO_3_^−^. Additionally, the presence of *CLC* reverse transporter function is determined based on whether both the gating amino acid of conserved residue (II) and the proton-transferring amino acid residue of conserved residue (III) are E (Glu). Thus, it can be inferred that Group e and Group f may function as non-selective channel proteins; Groups c and d may serve as Cl^−^ antiporters; Group g may act as a Cl^−^ channel protein; and Groups a/b may function as NO_3_^−^ antiporters. However, *MsCLC25* in Group a/b, lacking residue (III), might operate as a NO_3_^−^ channel protein. These findings are largely consistent with results from other species. For example, *AtCLC-a/b* function as NO_3_^−^/H^+^ antiporters, storing nitrate in vacuoles to provide nitrogen sources for plants [54,55]. *AtCLCc* and *AtCLCd* serve as Cl^−^ antiporters and play crucial roles in salt stress response [56,57,58]. This result further confirms the functional conservation of CLC proteins during plant evolution.

Transmembrane domain prediction revealed that MsCLCs proteins contain 7 to 19 transmembrane domains (Figure S1), structurally supporting their membrane protein characteristics. Numerous studies have demonstrated that the *CLC* gene family possesses conserved CBS and Voltage-gate CLC domains [29,30]. Our domain analysis of MsCLCs showed that each MsCLCs protein contains both CBS and Voltage-gate CLC domains, which are highly conserved across different species. Furthermore, different subclasses exhibit specific domain combinations (Figure 3C). These conserved domains provide crucial support for CLC proteins in ion transport processes [59], indicating their important role in ion homeostasis regulation.

Gene duplication events play a significant role in the expansion and evolution of a gene family [60]. Comparative analysis with other species reveals significant differences in the number of *CLC* gene family members among different plants. *Arabidopsis thaliana*, *Triticum aestivum, Solanum lycopersicum,* and *Nicotiana tabacum,* were found to contain 7, 23, 9, and 17 members, respectively [23,24,25,26], while this study identified 35 *MsCLC* genes in tetraploid alfalfa, a number significantly larger than most reported species. This difference in number is related not only to genome size but also to species-specific gene duplication patterns and evolutionary characteristics. In-depth analysis was conducted using the genome data of the tetraploid alfalfa cultivar ‘Xinjiangdaye’. The results revealed 48 segmental duplication events within the *CLC* gene family. These duplication events serve as the main driving force for both the expansion and evolution of this gene family. These segmental duplication events provide the material basis for the evolution of new functions by increasing gene copy number and enhancing genetic redundancy [45,46,61]. This process not only promotes the expansion of the alfalfa *CLC* gene family but also drives the evolution of new functions in polyploid alfalfa, thereby enhancing plant adaptability to environmental stress [47]. The Ka/Ks ratios for homologous gene pairs in the *MsCLCs* gene family were all less than 1, indicating they had undergone purifying selection. Additionally, synteny analysis identified 14, 6, 33, 40, 23, and 23 homologous gene pairs between *Medicago sativa* and *Arabidopsis thaliana*, *Oryza sativa, Medicago truncatula*, *Glycine max*, *Lotus japonicus*, and *Cicer arietinum*, respectively. The highest numbers of homologous genes were found between alfalfa and *Glycine max*/*Medicago truncatula*, further confirming their close phylogenetic relationship within Leguminosae and reflecting strong genome stability in leguminous plants during evolution, which has well preserved the *CLC* genes family.

Cis-acting element analysis helps understand and elucidate gene expression regulation patterns and reveals the function of related genes [62]. We predicted various elements responsive to biotic and abiotic stresses in the promoter regions of the *MsCLCs* gene family, including defense and stress response, low-temperature response, and hypoxia response elements, indicating that CLCs may play important roles in plant stress resistance and growth development. Except for MsCLC1, 3, 4, 10, 15, 16, and 26, all other members contain ABA-responsive elements (ABRE) in their promoter regions, suggesting they may participate in abiotic stress responses through ABA signaling. Gene expression patterns provide important evidence for elucidating gene functions. Therefore, we focused on analyzing the expression patterns of seven *MsCLC* genes under salt, drought, and ABA treatments (Figure 7). The results showed that ABA-induced gene expression levels were generally lower than those under salt and drought stresses. Under salt and drought stresses, these seven *MsCLC* genes were significantly upregulated in both root and leaf tissues, particularly the expression levels of *MsCLC2/18* from Group c were substantially higher than those of other subfamilies. Currently, the function of *CLC-c* has been widely documented; it enhances plant salt stress tolerance by regulating Cl^−^ accumulation [56]. Under salt stress, *Malus hupehensis MhCLC-c1* reduces intracellular Cl^−^ accumulation and decreases cell death [20]. Overexpression of *CsCLCc* in *Arabidopsis thaliana* reduces total Cl^−^ accumulation in roots and shoots, enhancing plant salt tolerance [63]. Therefore, the Group c subfamily (especially *MsCLC2/18*) may play a key role in alfalfa’s response to salt stress.

Subcellular localization demonstrated that *MsCLC18*, belonging to Group c, is a plasma membrane-localized protein (Figure 8), consistent with the localization of MhCLC-c1 protein [36]. However, some reports indicate that AtCLC-c is localized to the tonoplast [24], suggesting this difference may stem from species variation. Based on its root-to-leaf temporal expression pattern under NaCl stress, we propose that *MsCLC18* mediates Cl^−^ efflux from root cells during early stress, reducing Cl^−^ translocation to shoots. As stress persists, leaf expression increases to compensate for declining root efflux capacity. This spatiotemporally coordinated “root Cl^−^ efflux-leaf compensation transport” mechanism may effectively reduce Cl^−^ accumulation in aerial parts, thereby alleviating salt stress damage. The actual mechanism of *MsCLC18* gene action requires further investigation.

This study provides a systematic analysis of the *CLC* genes family in alfalfa and demonstrates that *MsCLC* genes are significantly induced by salt, drought, and ABA stresses. However, this research has certain limitations. At the functional level, although *MsCLC18* has been identified as a candidate chloride transporter, its Cl^−^ transport activity requires experimental validation. At the evolutionary level, the mechanisms governing the evolution and function of this gene family within the Medicago genus remain largely unclear. Future work will utilize CRISPR-Cas9 to generate knockout lines and Agrobacterium-mediated transformation for overexpression lines. The role of *MsCLC18* in Cl^−^ transport will be directly confirmed by non-invasive micro-test technology (NMT) for real-time monitoring of Cl^−^ flux and ion chromatography for measuring Cl^−^ content changes in different tissues under NaCl stress. and secondly, expanding research on the *CLC* gene family to other Medicago species to elucidate its evolutionary mechanisms and functional diversity. This study establishes a foundation for investigating how target genes function in salt-drought stress responses. It also provides important candidate gene resources for breeding stress-tolerant forage grasses.

## 4. Materials and Methods

### 4.1. Identification and Classification of the MsCLCs Gene Family

The whole-genome sequence and annotation files of “Xinjiangdaye” were downloaded from the FigShare database (https://figshare.com/, accessed on 1 April 2025); the CLC protein sequences of *Arabidopsis thaliana* were obtained from TAIR (https://www.arabidopsis.org/, accessed on 1 April 2025); and the CLC Hidden Markov Model (PF00654) was acquired from the InterPro database (https://www.ebi.ac.uk/interpro/, accessed on 1 April 2025) [64]. TBtools (v2.326) software was employed to perform BLASTP (E-value ≤ 1 × 10^−10^) and HMMER (E-value < 1 × 10^−5^) searches [65]. The protein family members identified by both methods were intersected, and the resulting protein sequences were validated using the NCBI Conserved Domains database (https://www.ncbi.nlm.nih.gov/Structure/cdd/wrpsb.cgi, accessed on 1 April 2025) [66], ultimately confirming the CLC family members in “Xinjiangdaye”.

Based on the reference genome annotation information of *Medicago sativa*, the chromosome locations were visualized using the Gene Location Visualize from GTF/GFF tool in TBtools (v2.326) software [67]. The identified CLC family members were subsequently named according to their positions on the chromosomes.

The amino acid length, molecular weight, isoelectric point, and other physicochemical properties of the MsCLC family members were predicted using the online ExPASy tool (https://web.expasy.org/protparam/, accessed on 7 April 2025) [68]. Subcellular localization predictions for the MsCLCs family members were performed using the Cell-PLoc online software (http://www.csbio.sjtu.edu.cn/bioinf/plant-multi/, accessed on 7 April 2025). Transmembrane domain predictions were conducted using the TMHMM Server v.2.0 (https://services.healthtech.dtu.dk/services/TMHMM-2.0/, accessed on 8 April 2025) [69]. Secondary structure predictions were performed using the SOPMA online tool (https://npsa-prabi.ibcp.fr/cgi-bin/npsa_automat.pl?page=npsa_sopma.html, accessed on 8 April 2025) [70].

### 4.2. Phylogenetic Analysis and Multiple Sequence Alignment Analysis of MsCLC Proteins

The CLC protein sequences for Arabidopsis and other plants are sourced from the TAIR and NCBI (https://www.ncbi.nlm.nih.gov/, accessed on 1 April 2025) (Table S4). phylogenetic trees of the CLC protein sequences from *Medicago sativa*, *Arabidopsis thaliana*, *Oryza sativa*, *Medicago truncatula*, *Glycine max*, *Lotus japonicus*, and *Cicer arietinum* was constructed using the MEGA (v11.0.10) software with the neighbor-joining (NJ) method and 1000 bootstrap replications [71]. The resulting NWK file was visualized using the ITOL website (https://itol.embl.de, accessed on 16 April 2025). Multiple sequence alignment of the MsCLC family members was performed using DNAMAN (v5.0) software, and the three conserved residues, GxGxPE (I), GKxGPxxH (II), and PxxGxLF (III), were analyzed using the MAST tool (https://meme-suite.org/meme/doc/mast.html, accessed on 20 April 2025).

### 4.3. Conserved Motifs, Domains, and Gene Structures of MsCLCs Gene Family

The motif composition of the *MsCLCs* gene family was analyzed using the MEME online tool (https://meme-suite.org/meme/tools/meme, accessed on 10 April 2025) [72]. Conserved domains were predicted using the NCBI CDD database (https://www.ncbi.nlm.nih.gov/cdd/, accessed on 10 April 2025). Visualization of conserved motifs, domains, and gene structures was performed using the Gene Structure View (Advanced) tool in TBtools (v2.326) software.

### 4.4. Gene Duplication Events and Colinearity Analysis of the MsCLCs Gene Family

Genome and GFF files for *Arabidopsis thaliana*, *Oryza sativa*, *Medicago truncatula*, *Glycine max*, *Lotus japonicus*, and *Cicer arietinum* were downloaded from the Ensembl Plants database (https://plants.ensembl.org/, accessed on 1 April 2025). The TBtools (v2.326) software was employed to analyze intra-species collinearity within alfalfa and inter-species collinearity between alfalfa and other species. To evaluate the evolutionary divergence among duplicated *MsCLC* genes, the Ka/Ks values were calculated using the KaKs Calculator implemented in TBtools (v2.326).

### 4.5. Promoter Cis-Regulatory Elements Analysis of MsCLCs Gene Family

The 2000 bp upstream sequences from the start codon of *MsCLC* genes were extracted using TBtools (v2.326). These sequences were subsequently submitted to the PlantCARE online website (https://bioinformatics.psb.ugent.be/webtools/plantcare/html/, accessed on 22 April 2025) for prediction of cis-acting elements in the promoter regions [73]. Visualization and heatmap analysis of the cis-acting elements were performed using the HeatMap tool in TBtools (v2.326). Additionally, different types of cis-acting elements were statistically analyzed, and the results were presented as a stacked bar chart generated with GraphPad Prism software (v10.1.2).

### 4.6. Analysis of the Expression Patterns of MsCLC Genes Under Different Abiotic Stress Treatments

This study used *Medicago sativa L*. ‘Xinjiangdaye’ as the material. Alfalfa seeds were sown in nutrient soil and cultivated at 25 °C under a 16 h light/8 h dark cycle for three weeks. The seedlings were then transferred to hydroponic containers with 1/2-strength Hoagland nutrient solution and grown for an additional week. Uniformly growing plants were selected and subjected to drought, salt, and ABA treatments. The treatments were as follows: four-week-old seedlings were exposed to drought stress (400 mmol·L^−1^ mannitol), salt stress (200 mmol·L^−1^ NaCl), and ABA treatment (10 μmol·L^−1^). Plant leaves and roots were collected at 0, 1, 3, 6, 9, 12, and 24 h (0 h served as the untreated control) for gene expression analysis. Each treatment included three biological replicates. All collected samples were immediately frozen in liquid nitrogen for total RNA extraction. Total RNA was extracted from the treated plant samples using the Difficult-to-Extract Total RNA Kit (Magen, Guangzhou, China) and reverse-transcribed into cDNA using the Reverse Transcription Kit (Vazyme, Nanjing, China). qRT-PCR was performed using gene-specific primers designed with Primer Premier 6.0 software (https://premierbiosoft.com, accessed on 15 April 2025). The Medicago actin gene was selected as the internal reference gene [74]. All primers used in this study are listed in Table S8. Reactions were carried out in a 20 μL volume using the LightCycler 480 II real-time PCR system (Roche, Shanghai, China). The relative expression levels of the MsCLCs genes in alfalfa under various abiotic stress conditions were determined using the 2^−ΔΔCT^ method proposed by Livak and Schmittgen [75]. Statistical significance was calculated using one-way ANOVA, with *p* < 0.05 considered highly significant.

### 4.7. Subcellular Localization Analysis

The pCAMBIA1300-eGFP vector, which was stored in our laboratory, was digested with BamH I and Sal I restriction enzymes (TakaRa, Dalian, China). The *MsCLC18* target fragment was ligated into the linearized vector using a homologous recombination kit (Vazyme, Nanjing, China), and the resulting product was subsequently transformed into Escherichia coli DH5α competent cells (Tsingke, Beijing, China). Positive clones verified by Sanger sequencing were further transformed into *Agrobacterium tumefaciens* GV3101 strain (Tsingke, Beijing, China). The plasma membrane localization plasmid AtPIP2A-mCherry was similarly transformed into the same bacterial strain [76]. *Agrobacterium tumefaciens* harboring the recombinant plasmids were cultured in LB liquid medium supplemented with kanamycin (50 mg/L) and rifampicin (25 mg/L) (Solarbio, Beijing, China) at 28 °C with shaking at 200 rpm for 24 h, after which the bacterial cells were collected. Sterile onion inner epidermal pieces of approximately 1 cm^2^ were prepared. A bacterial suspension was prepared using an infiltration buffer containing 1/2 MS (supplemented with 10 mM MgCl_2_, 10 mM MES, and 100 µM acetosyringone, pH 5.7). The optical density (OD) of the bacterial suspension was adjusted to 0.8–1.0. The prepared onion epidermal pieces were immersed in the bacterial suspension and incubated at 28 °C with shaking at 200 rpm for 45 min. Following incubation, the onion epidermis was removed, blotted dry on sterile filter paper, and placed on 1/2 MS solid medium for dark culture for 1–2 days. Prior to observation, the experimental samples were treated with 30% sucrose solution for 10 min to induce plasmolysis. Fluorescence signals were then observed using confocal laser scanning microscopy (Nikon, Tokyo, Japan) at excitation wavelengths of 488 nm and 560 nm, respectively [77].

## 5. Conclusions

Through genome-wide identification and analysis of the *MsCLCs* gene family in alfalfa, 35 members were identified and found to be unevenly distributed across 23 chromosomes. Phylogenetic and conserved residue analyses classified them into six subfamilies and major subclasses, with members of the same subclass sharing similar conserved domains and motifs. *MsCLCs* gene family expansion was primarily driven by forty-eight segmental duplication events, which enhance functional redundancy and provide evolutionary advantages for stress adaptation in tetraploid alfalfa. Expression analysis revealed critical roles of *MsCLC* genes in salt and drought stress responses, with *MsCLC18* from Group c showing significant induction in both roots and leaves, displaying a root-to-leaf temporal expression pattern. Subcellular localization confirmed its plasma membrane localization, identifying it as a key player in stress response. These findings establish a foundation for further elucidating the molecular mechanisms of *MsCLC* genes in abiotic stresses and provide theoretical support for gene mining and utilization in stress-resistant molecular breeding of alfalfa.

## Data Availability

The original contributions presented in this study are included in the article/Supplementary Material. Further inquiries can be directed to the corresponding author(s).

## Supplementary Materials

The following supporting information can be downloaded at: https://www.mdpi.com/article/10.3390/ijms262311442/s1.
