# Supplementary material for: Genome-Wide Identification and Functional Divergence of the Chloride Channel (CLC) Gene Family in Autotetraploid Alfalfa (Medicago sativa L.)
Source: Int J Mol Sci. 2025 Nov 26;26(23):11442. doi: 10.3390/ijms262311442 (PMC12692330; doi:10.3390/ijms262311442)
Supplement: Supplementary file 1 [file ijms-26-11442-s001.zip › ijms-3986418-supplementary/Supplementry Figures/Figure S1.Transmembrane domains of MsCLCs.pdf]

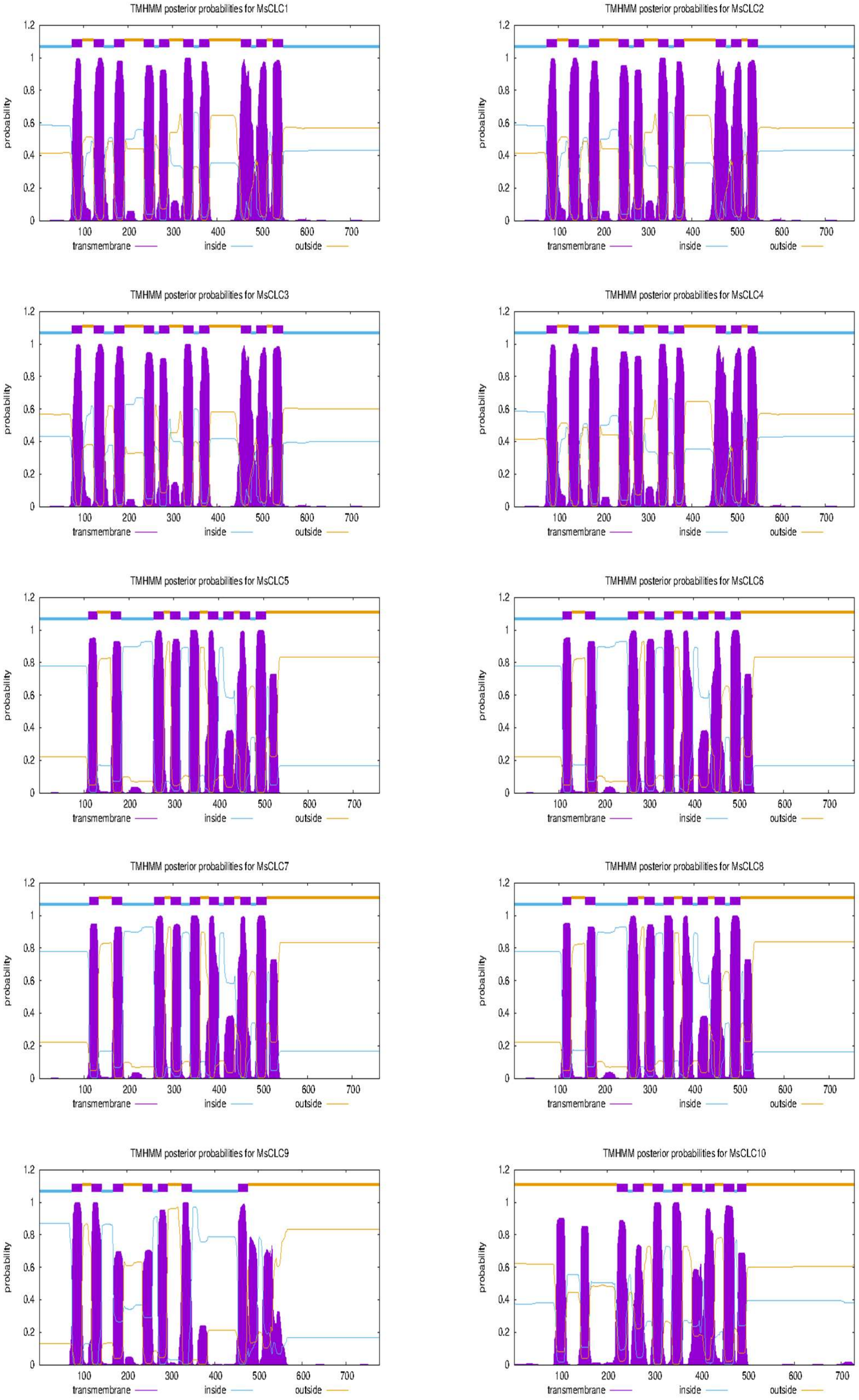
TMHMM posterior probabilities for MsCLC1
TMHMM posterior probabilities for MsCLC2
TMHMM posterior probabilities for MsCLC3
TMHMM posterior probabilities for MsCLC4
TMHMM posterior probabilities for MsCLC5
TMHMM posterior probabilities for MsCLC6
TMHMM posterior probabilities for MsCLC7
TMHMM posterior probabilities for MsCLC8
TMHMM posterior probabilities for MsCLC9
TMHMM posterior probabilities for MsCLC10
probability
transmembrane
inside
outside

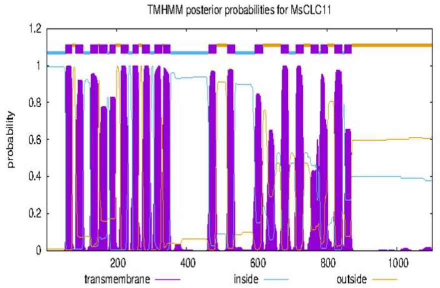
TMHMM posterior probabilities for MsCLC11
probability
transmembrane
inside
outside

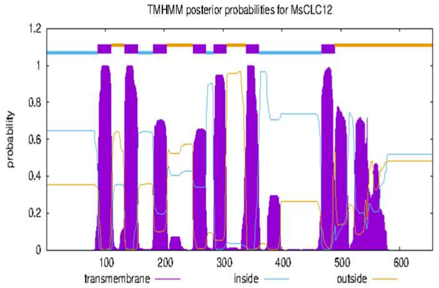
TMHMM posterior probabilities for MsCLC12
probability
transmembrane
inside
outside

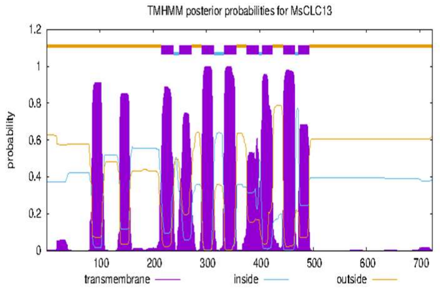
TMHMM posterior probabilities for MsCLC13
probability
transmembrane
inside
outside

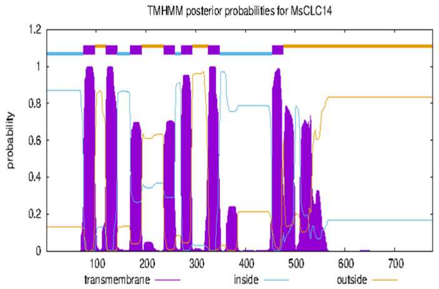
TMHMM posterior probabilities for MsCLC14
probability
transmembrane
inside
outside

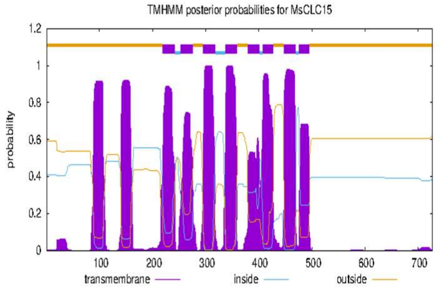
TMHMM posterior probabilities for MsCLC15
probability
transmembrane
inside
outside

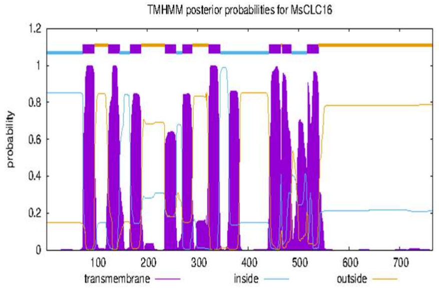
TMHMM posterior probabilities for MsCLC16
probability
transmembrane
inside
outside

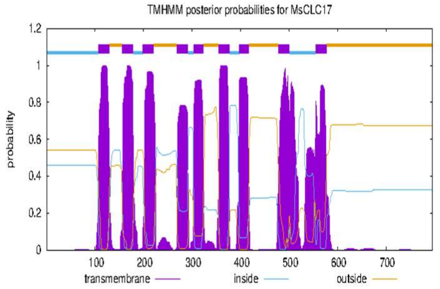
TMHMM posterior probabilities for MsCLC17
probability
transmembrane
inside
outside

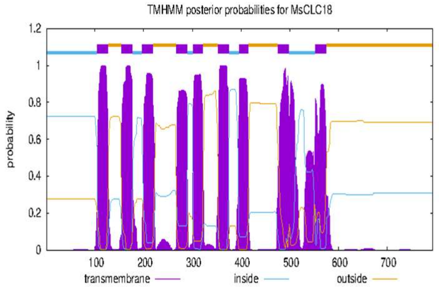
TMHMM posterior probabilities for MsCLC18
probability
transmembrane
inside
outside

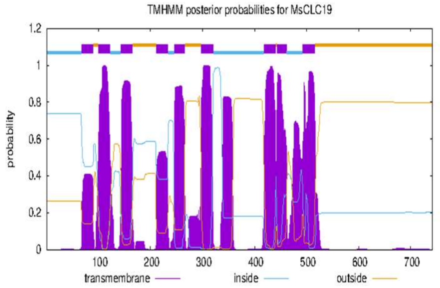
TMHMM posterior probabilities for MsCLC19
probability
transmembrane
inside
outside

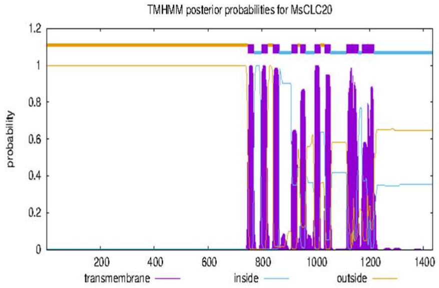
TMHMM posterior probabilities for MsCLC20
probability
transmembrane
inside
outside

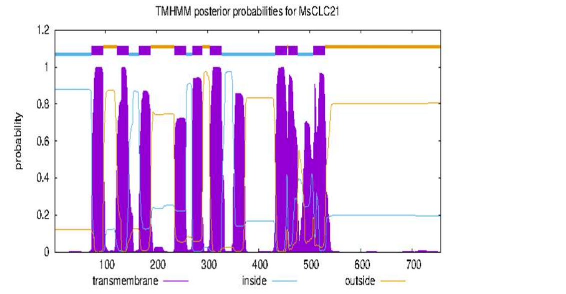
TMHMM posterior probabilities for MsCLC21
probability
transmembrane
inside
outside

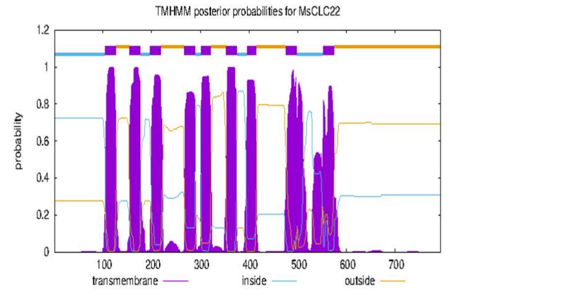
TMHMM posterior probabilities for MsCLC22
probability
transmembrane
inside
outside

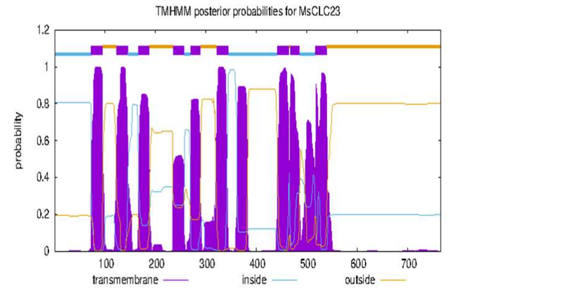
TMHMM posterior probabilities for MsCLC23
probability
transmembrane
inside
outside

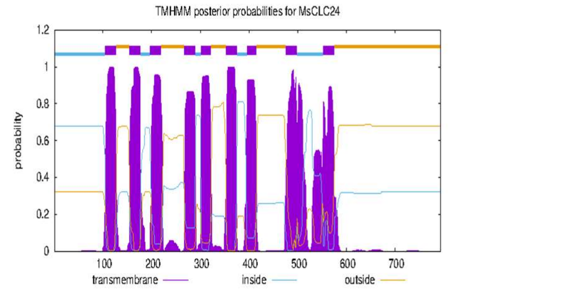
TMHMM posterior probabilities for MsCLC24
probability
transmembrane
inside
outside

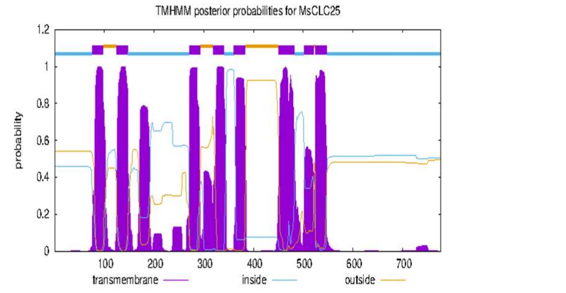
TMHMM posterior probabilities for MsCLC25
probability
transmembrane
inside
outside

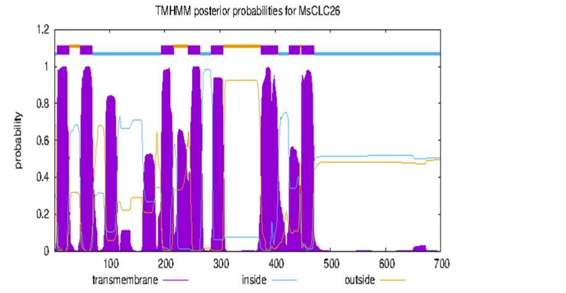
TMHMM posterior probabilities for MsCLC26
probability
transmembrane
inside
outside

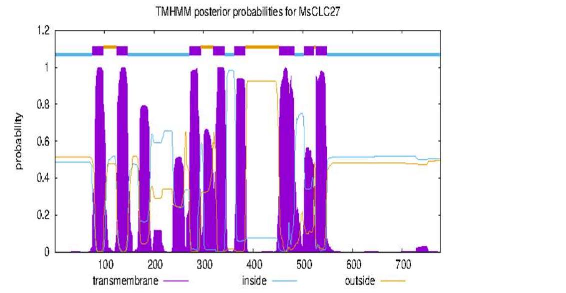
TMHMM posterior probabilities for MsCLC27
probability
transmembrane
inside
outside

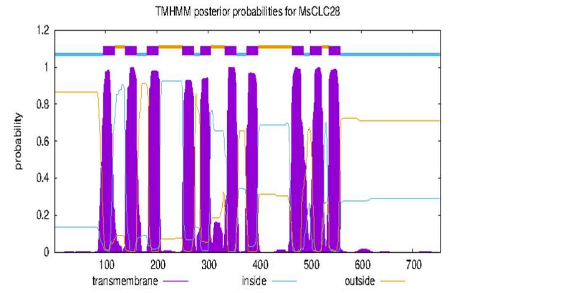
TMHMM posterior probabilities for MsCLC28
probability
transmembrane
inside
outside

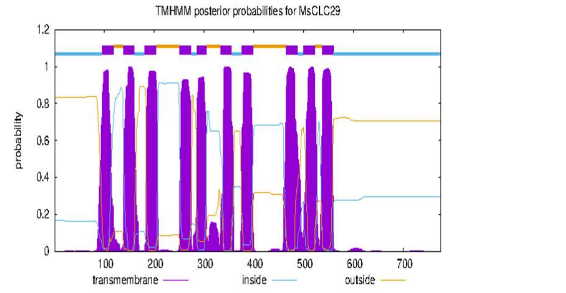
TMHMM posterior probabilities for MsCLC29
probability
transmembrane
inside
outside

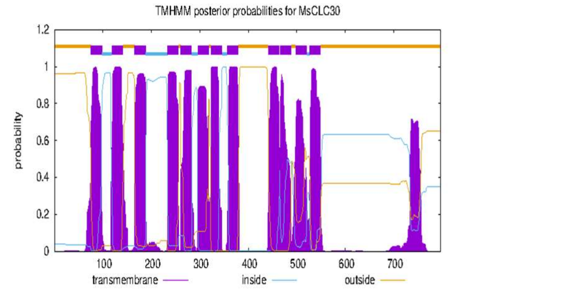
TMHMM posterior probabilities for MsCLC30
probability
transmembrane
inside
outside

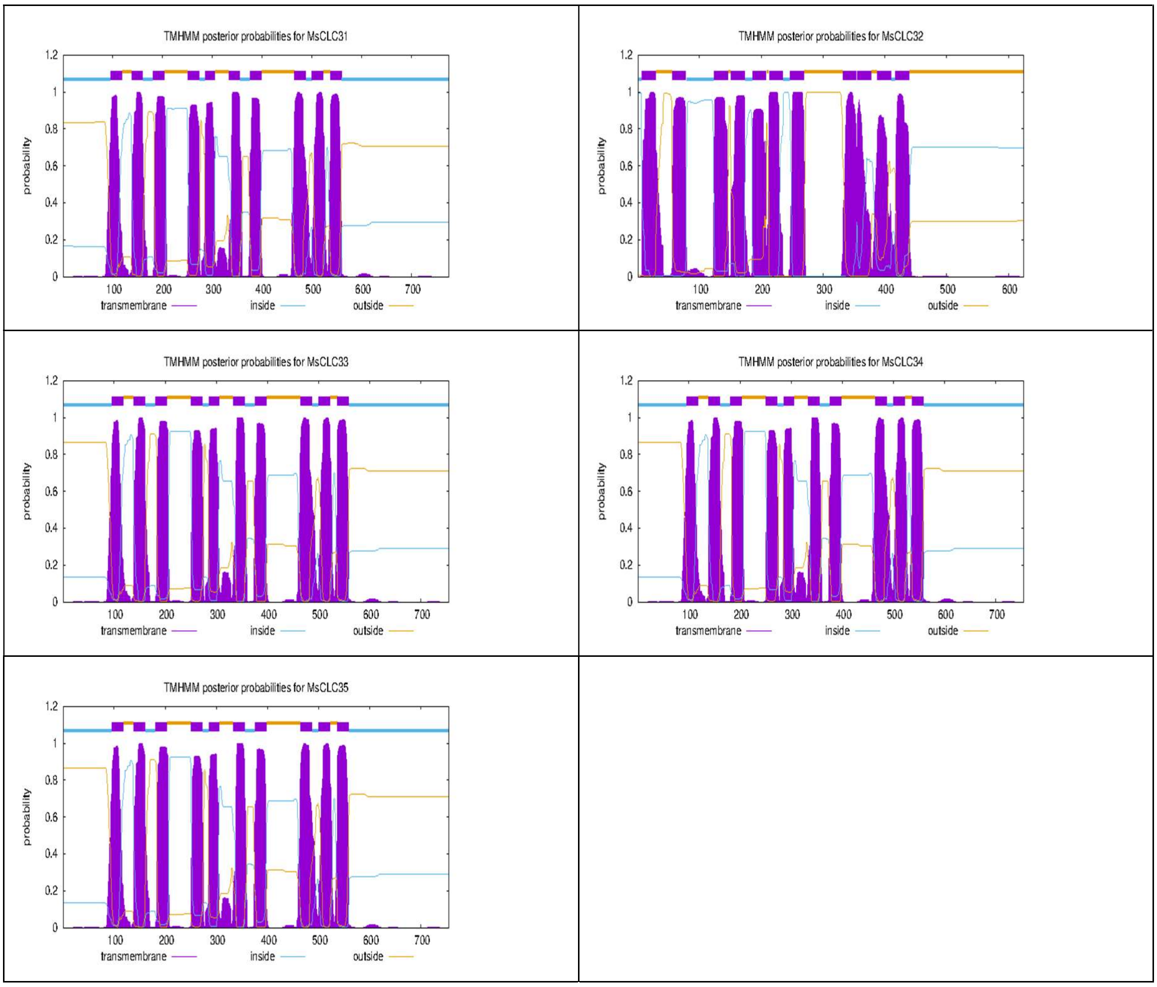

Figure S1. The predicted results of the transmembrane structure of alfalfa MsCLCs are represented by three colored lines. The yellow line represents the lateral region, the blue line represents the medial region, and the purple line represents the transmembrane domain.
