# Supplementary material for: Genome-Wide Identification and Functional Divergence of the Chloride Channel (CLC) Gene Family in Autotetraploid Alfalfa (Medicago sativa L.)
Source: Int J Mol Sci. 2025 Nov 26;26(23):11442. doi: 10.3390/ijms262311442 (PMC12692330; doi:10.3390/ijms262311442)
Supplement: Supplementary file 1 [file ijms-26-11442-s001.zip › ijms-3986418-supplementary/Supplementry Figures/Figure S2.This is the prediction of the secondary structure of alfalfa MsCLCs.pdf]

MsCLC1

MsCLC2

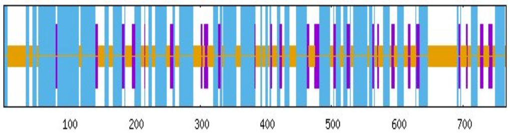

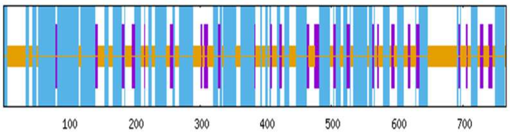

MsCLC3

MsCLC4

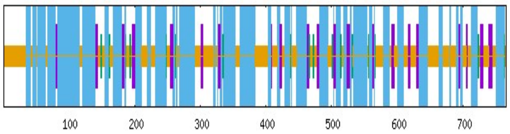

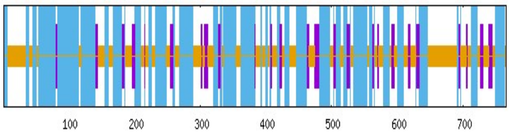

MsCLC5

MsCLC6

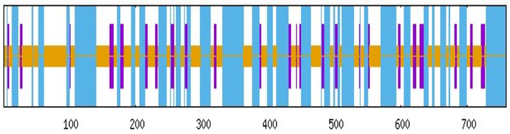

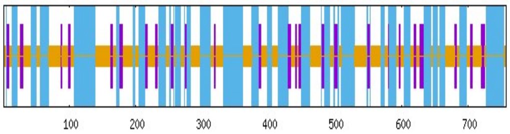

MsCLC7

MsCLC8

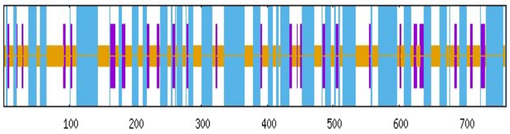

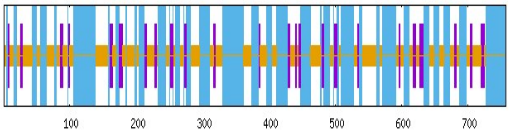

MsCLC9

MsCLC10

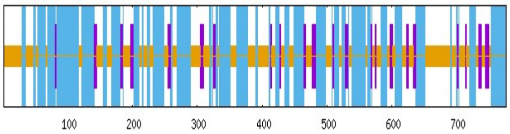

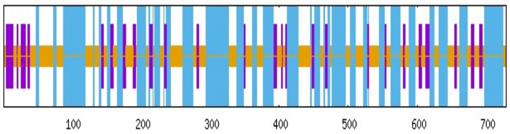

MsCLC11

MsCLC12

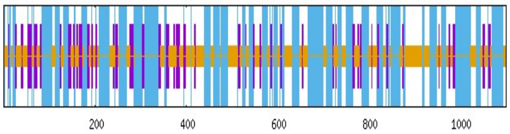

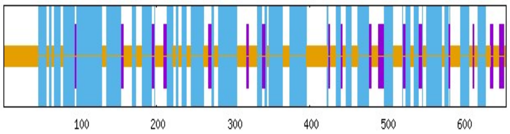

MsCLC13

MsCLC14

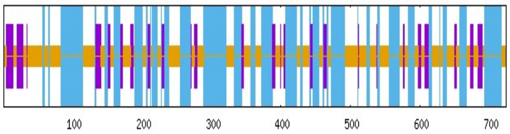

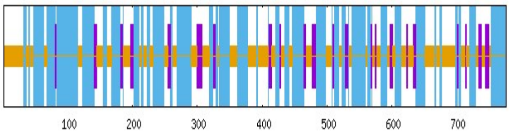

MsCLC15

MsCLC16

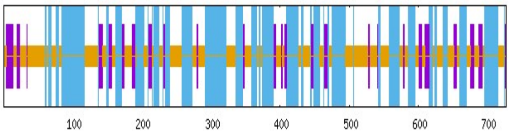

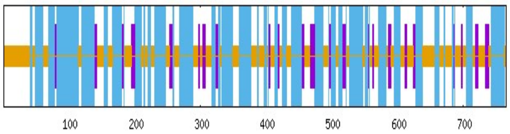

MsCLC17

MsCLC18

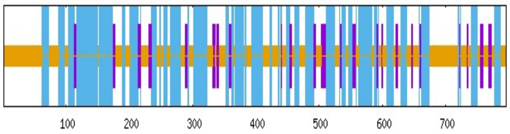

MsCLC19

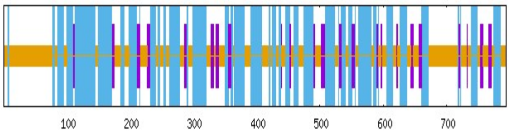

MsCLC20

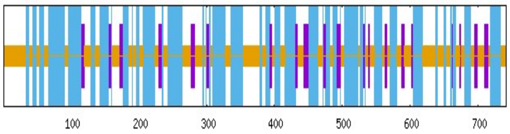

MsCLC21

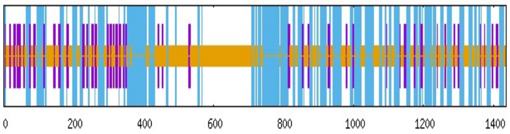

MsCLC22

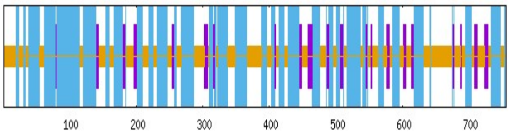

MsCLC23

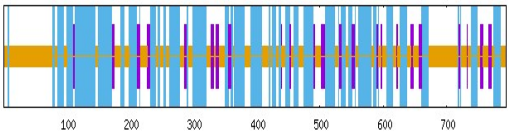

MsCLC24

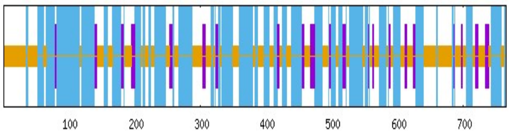

MsCLC25

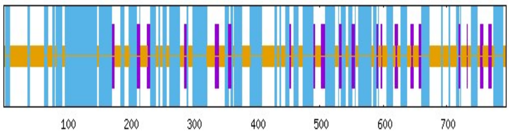

MsCLC26

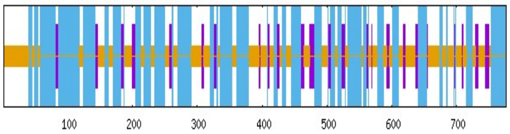

MsCLC27

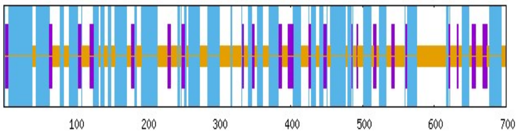

MsCLC28

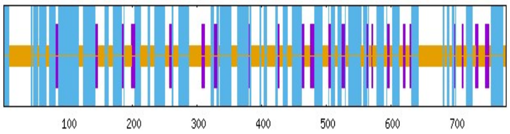

MsCLC29

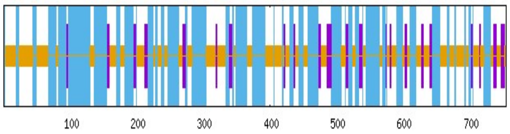

MsCLC30

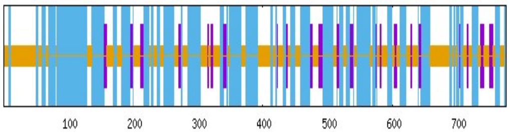

MsCLC31

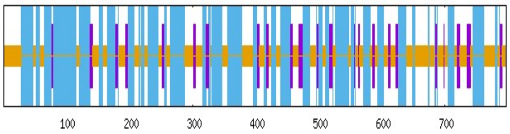

MsCLC32

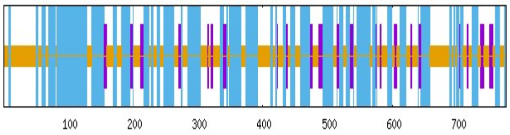

MsCLC33

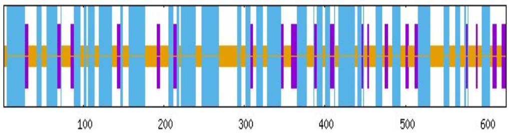

MsCLC34

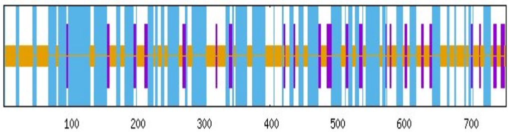

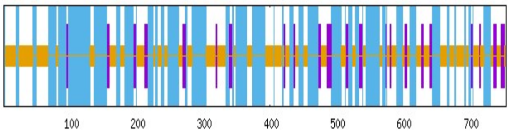

MsCLC35

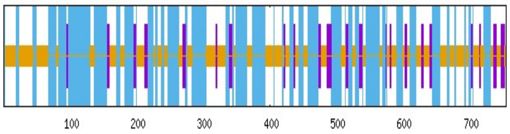

Figure S2. This is the prediction of the secondary structure of alfalfa MsCLCs, the colored part is the location of the corresponding secondary structure, blue represents α helix, purple represents extended chain, and yellow represents irregular curling.
