# Supplementary material for: Genome-Wide Identification and Functional Divergence of the Chloride Channel (CLC) Gene Family in Autotetraploid Alfalfa (Medicago sativa L.)
Source: Int J Mol Sci. 2025 Nov 26;26(23):11442. doi: 10.3390/ijms262311442 (PMC12692330; doi:10.3390/ijms262311442)
Supplement: Supplementary file 1 [file ijms-26-11442-s001.zip › ijms-3986418-supplementary/Supplementry Figures/Figure S3.Multiple sequence alignment of the MsCLCs family amino acid sequences.pdf]

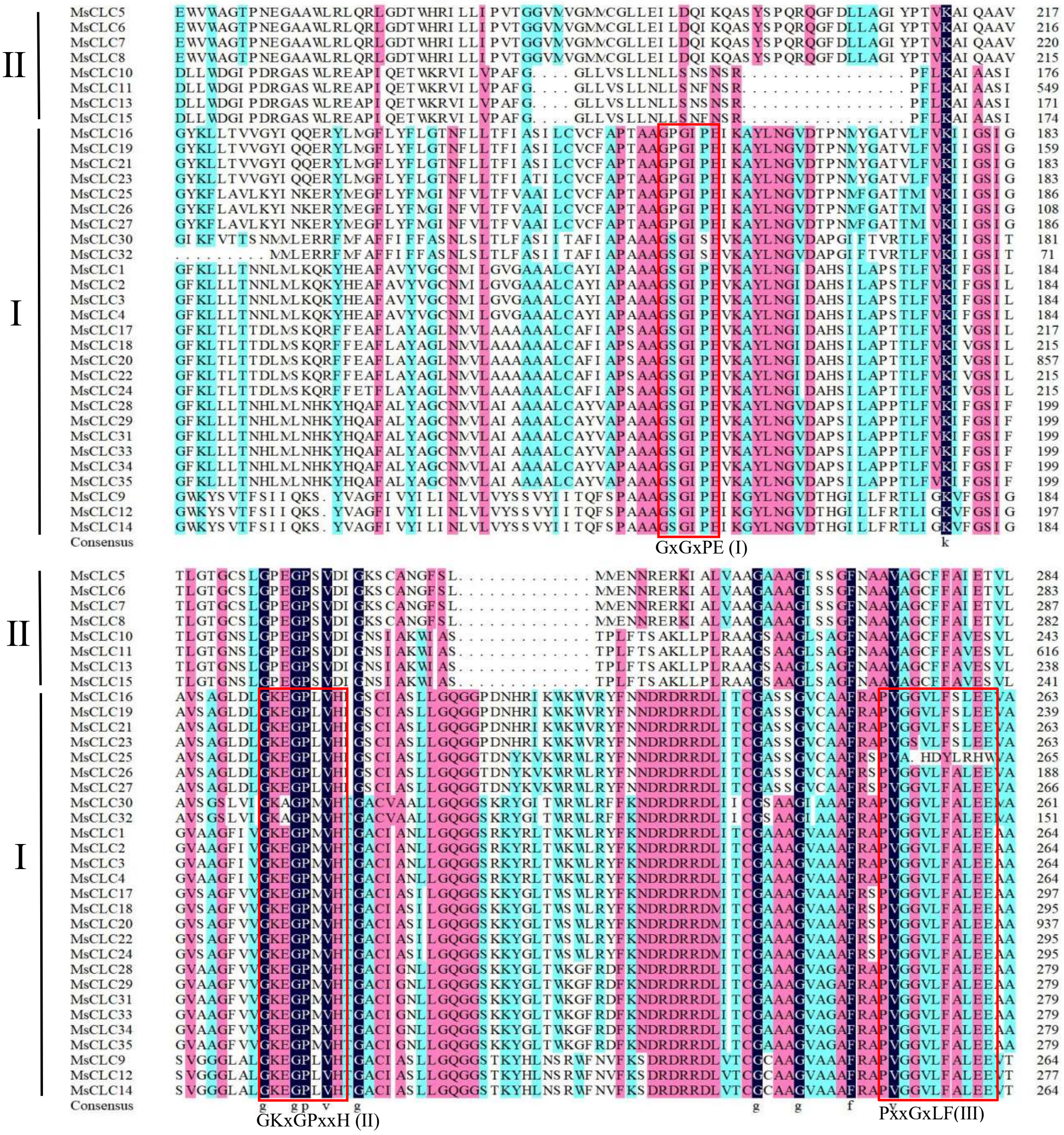

Figure S3. Multiple sequence alignment of the MsCLCs family amino acid sequences. Amino acid residues marked in different colors represent their degree of conservation. Black lines indicate the positions of completely conserved motifs. Pink represents a 70% conservation rate, and blue represents a 60% conservation rate.
