# Supplementary material for: Genome-Wide Identification and Functional Divergence of the Chloride Channel (CLC) Gene Family in Autotetraploid Alfalfa (Medicago sativa L.)
Source: Int J Mol Sci. 2025 Nov 26;26(23):11442. doi: 10.3390/ijms262311442 (PMC12692330; doi:10.3390/ijms262311442)
Supplement: Supplementary file 1 [file ijms-26-11442-s001.zip › ijms-3986418-supplementary/Supplementry Figures/Figure S4.The motif symbol of MsCLCs shares a sequence with the motif.pdf]

A

Motif	Symbol	Motif Consensus
1.		CGAAAGVAAAFRAPVGGVLFALEEVASWW
2.		LGAASFLGGTMRMTVSLCVILLELTNBLLLLP
3.		GFVLGKEGPMVHTGACIASLLGQGGSKKY
4.		NFQCPPNHYNDLASLFFTTNDDAIRNLFS
5.		NTSPYTVVETMSLAKAAILFRZLGLRHLLVVP
6.		VADCFNKGVYDQILELKGLPYLEAHAEPYMRN
7.		AAALCAYIAPAAAGSGIPEVKAYLNGVDAPSI
8.		TSQLAJVGANVCPIESLDYEIIENDLFKQDWR
9.		FSGIEKVGNIVHVLKVTTHNGFPVIDEPP
10.		RSALLWRTFFTTAVVAVVLRAFIZFCKSGKCG
11.		YFLGIITYGIAVPSGLFIPVILAGAAYGRLVG
12.		FKJJLAMIISLLTSCCSFGLPWLAKCIPC
13.		GLTWKWLRYFKNDRDRRDLIT
14.		ENIAGFKLLLTNHLMLKQRYHEAFLYYAGCNM
15.		KPGSGKGIKLEDLDITEEEMEMYVDLHPJ
16.		KASGRPPIVGILTRHDFMAEHILGLFPHLASS
17.		GSLYNYLVDKVLRTYNJINEK
18.		RAKVZIVQYVVLKWTFALLIGLGTGLVAFFNN
19.		FGEGGLIMFDVNSVKPAYSVPDJLPVIFJ
20.		SEAPELCGLVLRSHLJVJLKHKA

B

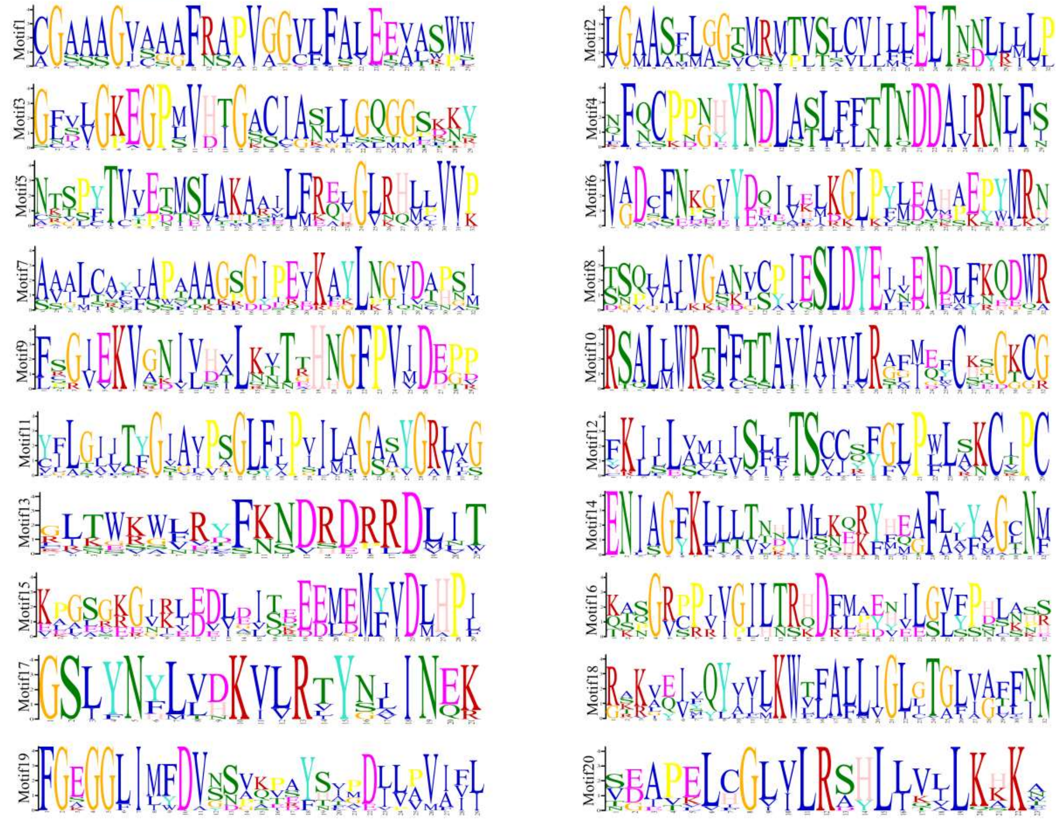

Figure S4. (A) The motif symbol of MsCLCs shares a sequence with the motif. The different motif symbols are displayed on the left, and the protein sequences of all the motifs of MsCLCs are displayed on the right. (B) Motif identifier map of conserved consensus sequences of MsCLCs. The total height of each column indicates the sequence conservatism of that locus; The height of each residue letter reflects the relative occurrence frequency of the corresponding residue (color illustration). The bottom ruler is used to compare the lengths of different proteins.
