# Supplementary material for: Genome-Wide Identification and Functional Divergence of the Chloride Channel (CLC) Gene Family in Autotetraploid Alfalfa (Medicago sativa L.)
Source: Int J Mol Sci. 2025 Nov 26;26(23):11442. doi: 10.3390/ijms262311442 (PMC12692330; doi:10.3390/ijms262311442)
Supplement: Supplementary file 1 [file ijms-26-11442-s001.zip › ijms-3986418-supplementary/Supplementry Figures/Figure S5.Analysis of cis-acting elements of promoters of MsCLCs genes.pdf]

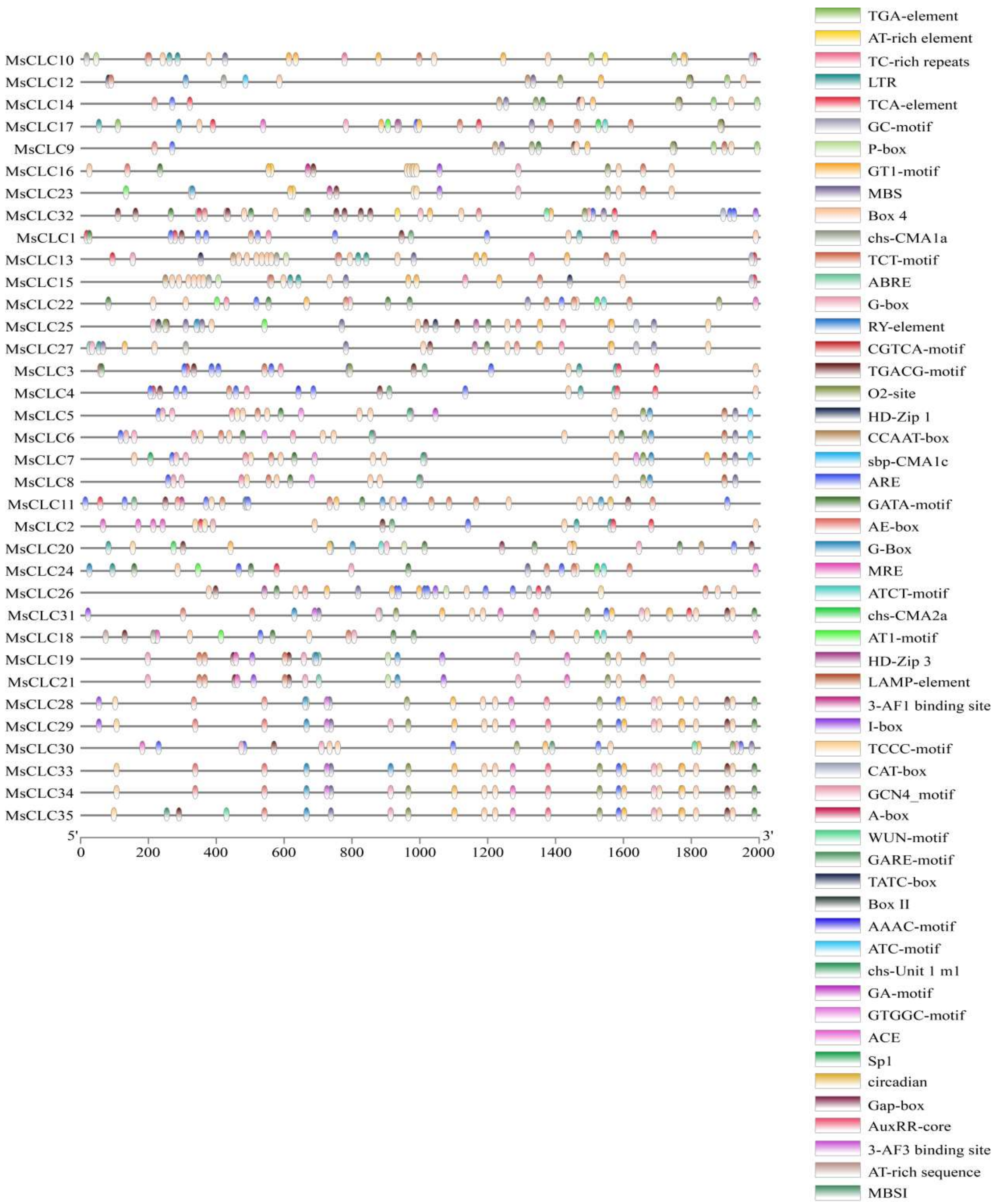

Figure S5. Analysis of cis-acting elements of promoters of *MsCLCs* genes. The different types of cis-acting elements are represented by different shapes and colors.
