# Supplementary material for: Genome-Wide Identification and Functional Divergence of the Chloride Channel (CLC) Gene Family in Autotetraploid Alfalfa (Medicago sativa L.)
Source: Int J Mol Sci. 2025 Nov 26;26(23):11442. doi: 10.3390/ijms262311442 (PMC12692330; doi:10.3390/ijms262311442)
Supplement: Supplementary file 1 [file ijms-26-11442-s001.zip › ijms-3986418-supplementary/Supplementry Figures/Figure S6.Expression of MsCLC gene under salt stress.pdf]

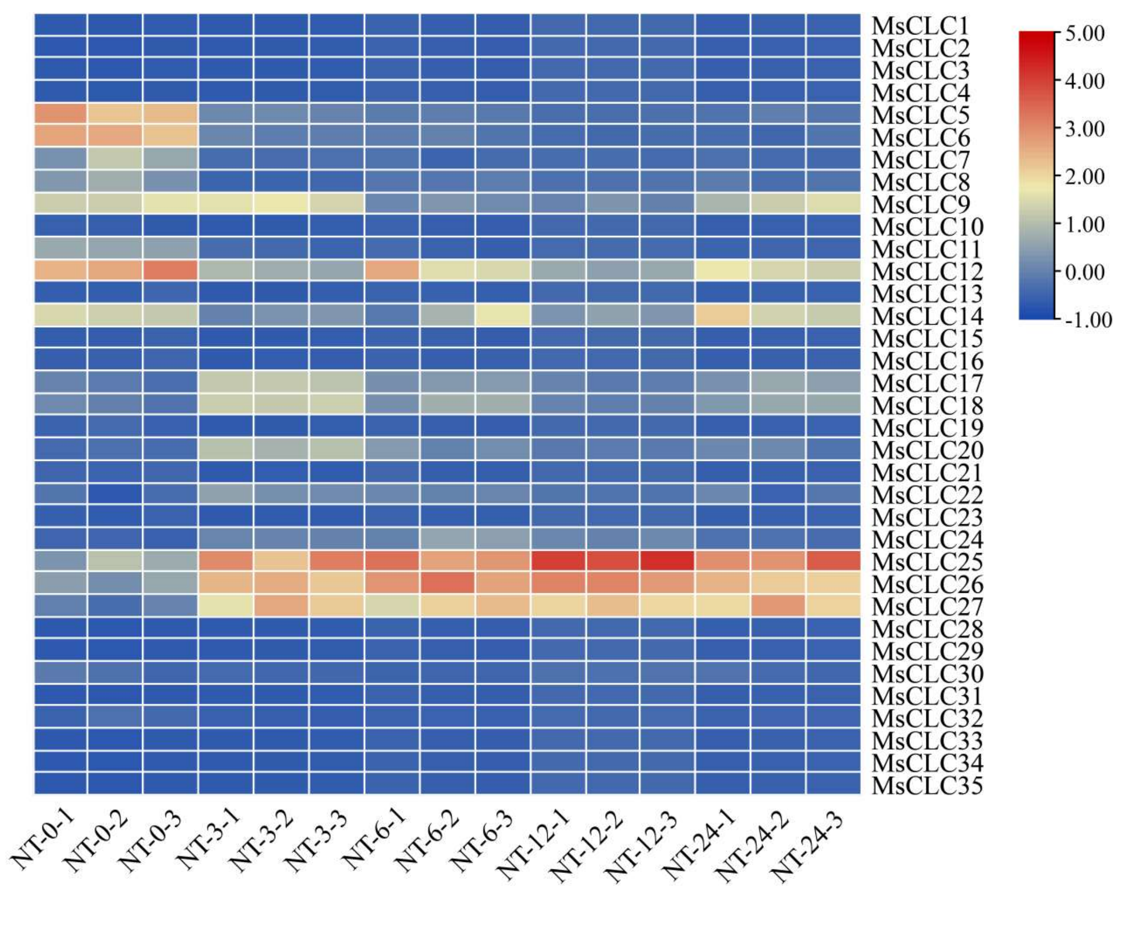

Figure S6. Expression of MsCLC gene under salt stress. Color bars from blue to red indicate relative expression levels from low to high.
